# Lessons in cognitive unloading, skills mixing, flattened hierarchy and organisational agility from the Nightingale Hospital London during the first wave of the *SARS-CoV-2* pandemic

**DOI:** 10.1136/bmjoq-2021-001415

**Published:** 2021-07-22

**Authors:** George Benjamin Collins, Nikhil Ahluwalia, Lynne Arrol, Natalie Forrest, Alan McGlennan, Ben O'Brien, Alastair Proudfoot, Matthew Trainer, Richard Schilling, Eamonn Sullivan, Mark Westwood, Andrew Wragg, Charles Knight

**Affiliations:** 1 Division of Medicine, University College London, London, UK; 2 Department of Cardiology, Barts Health NHS Trust, London, UK; 3 Department of Cardiac Electrophysiology, Barts Health NHS Trust, London, UK; 4 Department of Clinical Governance, Barts Health NHS Trust, London, UK; 5 Senior Responsible Owner, New Hospital Programme, Department of Health and Social Care, London, UK; 6 Chief Executive, Barnet and Chase Farm Hospitals NHS Trust, London, UK; 7 Department of Anaesthetics, Royal Free London NHS Foundation Trust, London, UK; 8 Medical Director, Barnet and Chase Farm Hospitals NHS Trust, London, UK; 9 Department of Intensive Care Medicine, Barts Health NHS Trust, London, UK; 10 Department of Anaesthetics, German Heart Center, Charité Medical Faculty, Berlin, Germany; 11 Department of Intensive Care, Barts Health NHS Trust, London, UK; 12 Queen Mary University of London, London, UK; 13 Chief Executive, Oxleas NHS Foundation Trust, Dartford, UK; 14 Chief Nurse (testing), NHS Test and Trace, Department of Health and Social Care, London, UK; 15 Chief Nurse, Royal Berkshire NHS Foundation Trust, Reading, UK; 16 Department of Cardiac Imaging, Barts Health NHS Trust, London, UK; 17 Department of Interventional Cardiology, Barts Health NHS Trust, London, UK

**Keywords:** clinical governance, continuous quality improvement, COVID-19, healthcare quality improvement, organisational culture

## Introduction

The Nightingale Hospital London (NHL) was the first of seven new UK National Health Service (NHS) hospitals designed to address the potential shortfall in critical care capacity caused by the rapidly escalating first wave of the *SARS-CoV-2* pandemic. When scenes of hospitals at risk of saturation were being broadcast internationally, the initial NHL mandate was to deliver a large-scale intensive care unit (ICU) for up to 4000 ventilated patients to increase the capacity of London’s existing hospitals. Before this, ICU bed capacity in London and England was 839 and 3766, respectively.^1^ Healthcare projects of this pace, scale or complexity are usually overseen by experienced teams over many years, and within specifically designed environments and timetables.^2^ However, the NHL was constructed in an events centre, and started accepting patients within weeks of conception. The newly formed NHL leadership team was redeployed from different hospitals across London at a time of unprecedented national uncertainty, before disease-modifying COVID-19 therapies were approved and when staff in existing hospitals were preparing ICU expansions of their own.^3^

This unique mandate resulted in the NHL being exposed to a level of potential risk and vulnerability to human error that healthcare services have worked to reduce in recent years.^4 5^ Fortunately, due to lockdown restrictions and ICU expansion in existing hospitals, only 54 patients were admitted to the NHL. Therefore, in comparison to forecasts, existing hospitals and its potential capacity, the overall contribution of the NHL to the ICU COVID-19 response in London was relatively small. Importantly, however, clinical outcomes were comparable to existing hospitals. NHL patient mortality was 48.1% compared with 47.7% nationally, and the requirements for, and duration of, organ support were also similar.^6^ Although its true potential was fortunately never reached, the NHL did fulfil its role in urgently providing a safe ICU facility that was capable of rapid expansion if required, had existing hospitals lacked the necessary capacity as originally predicted.

In doing so, the NHL was a rare ‘natural experiment’ for the NHS, from which important and potentially transferable lessons can be learnt and shared. Rapidly creating a safe, sizeable and scalable ICU in a non-clinical environment presented unique challenges that required novel and often emergent solutions.^7^ This mandate produced an environment of anticipation, adversity, unfamiliarity and uncertainty, to which creativity and innovation were necessary and natural responses.^2^ While it is important to recognise that important changes took place in existing hospitals, the unusual circumstances of care at the NHL presented a unique set of challenges, solutions and innovations. The authors believe that many of the lessons from the NHL could be relevant to existing hospitals during non-pandemic times, and that sharing these could be the catalyst to support healthcare changemakers to investigate or implement similar reforms of their own.

On that basis, this article discusses the context of care and the challenges faced at the NHL, alongside important lessons that could be taken away and developed. Innovations and lessons from all aspects of care are included, with particular focus on those considered to be most relevant, transferable or impactful. Being both a recent and ‘natural experiment’, negative controls, prospective protocols, experimental end points and long-term analyses are inevitably unavailable. As a result, the discussion is unavoidably descriptive, inferential and based on routinely collected health data. However, written by the complete leadership team, the intention is to convey a range of views from different perspectives and backgrounds, which together provide a consensus position on the realities of care at the NHL.

## Task overview

The only venue that could feasibly provide the capacity needed to urgently address the potential shortfall in London’s ICU provision was the ExCeL events centre in Newham, East London. To meet the NHS legal requirements for organisational accountability, the NHL was overseen by Barts Health NHS Trust—the second largest organisational unit of healthcare provision in the UK, which also manages five nearby hospitals.^8^ The initial mandate was to provide a large-scale ICU prepared for rapid expansion, to ensure the availability of beds for all patients with COVID-19 requiring invasive respiratory support. With that in mind, using a collaboration between military personnel, clinicians and healthcare managers, the NHL was constructed in 9 days. After 2 months in operation, the NHL was transformed into a hospital in waiting, and then into hibernation. These abrupt changes in mandate required a level of agility and preparedness that established hospitals may be less accustomed to during non-pandemic times.^9^

## Staff recruitment

Without existing employees or workforce plans, an early concern was staff recruitment. Established hospitals were expanding their ICU provision in parallel, and reporting pressures in staff sickness and bed occupancy of their own.^3^ To avoid compromising existing NHS services, the pool from which the NHL could recruit was small and atypical. This required new staffing models, and many of the usual rules of appointment and duty allocation were necessarily rewritten. The majority of NHL recruits were self-selecting volunteers or contacts of staff. Many could only join because their routine responsibilities were suspended. For example, academics pursuing postponed non-COVID-19 research, surgeons whose theatre lists were deferred and even non-medical staff such as airline stewards, whose flights were rescheduled. The result was a unique and disparate group of self-selecting self-motivated staff from different backgrounds, hospitals and professions. While presenting its own challenges, this also led to an originality and diversity in thought which—combined with the general sense of urgency and absence of traditional ‘expertise’, both a result of the pathogenicity, transmissibility and novelty of COVID-19—led to new staffing models, and a heightened sense of entrepreneurship at the NHL. For example, the mortuary was managed by an orthopaedic surgeon, whose elective surgery was postponed. Many of the senior management team were cardiologists rather than intensivists, whose elective work had been cancelled, but who had important transferable skills. Finally, because ICU specialists were in short supply, steps were taken to reduce the risk of ‘cognitive overload’—where focus and task execution are impaired by excessive sensory input and service demands.^10 11^ For example, at the NHL, an updated prescription chart, including pre-written prescriptions for gastroprotection, anticoagulation and hydration, was implemented within days of conception. In existing hospitals during non-pandemic times, this can take weeks, if not months.^12^

## Workforce deconstruction and duty reallocation

Due to the absence of existing staff and similar staffing needs in established hospitals, some of the clinical workforce available to the NHL was not specifically ICU trained. Further innovations were therefore needed to maximise the use of ICU staff, and redistribute selected duties to other team members. New recruits were upskilled to support the ICU workload and expand the workforce. For example, the duties of ‘Clinical Support Workers’ (CSWs)—a new role created at the NHL combining the responsibilities of healthcare assistants and ICU nurses—were designed for non-ICU and even non-clinical staff. Airline stewards, podiatrists, optometrists and school nurses, working as CSWs, were trained to record observations, change pre-prepared infusions, maintain patient hygiene and assess pressure areas. To further support cognitive unloading of ICU nurses, certain tasks were transferred to more available staff. Syringe infusions were prepared by pharmacists, doctors performed blood gases and venepuncture and physiotherapy-led ‘proning teams’ performed this evidence-based but labour-intensive intervention. Similarly, to support ICU registrars, point-of-care ultrasonography was performed by radiologists, non-ICU clinicians were upskilled as ward doctors and central venous and arterial cannulations were undertaken by a cardiologist-led ‘lines team’. Finally, to relieve all clinical staff of an administrative burden, the incident report form was shortened, and instead completed by a new ward-based governance role—the ‘Bedside Learning Coordinator’ (BLC)—which is discussed below. Overall, by reallocating duties away from staff in short supply, ICU nurses and doctors could focus on their specialist non-transferable skills, including airway management, patient oversight, decision-making and team leadership. With that in mind, at the NHL, the workforce model was that CSWs were responsible for 1 patient each, ICU nurses for 6 and ICU consultants for 30—significantly more diluted ratios than ICUs traditionally permit.^13^

## Staff training and welfare

From the start, the high-pressure environment of the NHL was expected to negatively affect staff well-being. Cognitive unloading therefore became an institutional priority extending to all staff, both on and off the wards. A well-being directorate was established alongside normal hospital directorates, including a Director of Well-being on the Leadership Board and a committee of psychologists, occupational health specialists, human resource managers and a psychiatrist. This was in anticipation of unfamiliar working conditions, new clinical roles, high COVID-19 mortality, and the novelty, urgency and anticipated trajectory of the pandemic.

At induction, employees underwent ‘psychological PPE’ training, a novel educational programme created at the NHL to educate staff in self-reflection, anxiety management and mindfulness.^14^ Before each shift, staff were paired with a colleague to monitor for signs of distress. Psychological support was available, including pathways for onward referral if indicated. Physical well-being was supported by complimentary food, drink, parking and accommodation. Finally, a workforce support desk was established in rest areas to address clerical issues, including contracts, pay, transport and accommodation.

Medical education and induction were based at the repurposed O2 entertainment arena, a nearby events venue. Induction was stripped down, clinically orientated and updated daily to align with changes in hospital mandate, service provision and clinical need. The day before their first shift, ‘day zero’ simulation familiarised new staff with NHL bedspaces, equipment, protocols and working patterns. Clinical and non-clinical staff were given an online ‘Nightingale NHS ePortfolio’ to record well-being plans, shift logs, reflective practice and learning outcomes.^15^ This was linked to a new interdisciplinary competency framework, written at the NHL, which for the first time in NHS history defined an agreed learning curriculum that applied equally to all staff, regardless of their role, training or ambitions.^15^ While difficult to quantify and likely multifactorial, the success of these education and well-being initiatives is supported by a staff sickness rate at the NHL of under 2%—one-tenth of that contemporaneously reported by existing NHS hospitals.^16^ Other factors, such as staff demographics and social separation whilst at the NHL, may also have contributed to this.

## Flattened hierarchy

By their nature, shortcomings in clinical expertise are inevitable during a novel pandemic.^17^ As a result, traditional hierarchies based on experience and expertise become less reliable. At the NHL, this was exacerbated by the unusual working environment, and heightened sense of urgency and anticipation. The result was a weakening of traditional hierarchies, and emergence of a so-called ‘flattened hierarchy’.^18^ This permitted the delegation of tasks ‘down’ to the most invested stakeholder, rather than ‘up’ to the most responsible. This model, which arose spontaneously, benefited from the physical layout of the ExCeL centre, where an open-plan management area was constructed in the large exhibition hall. Lacking segregated offices, this served as a shared but socially distanced workspace, where senior managers could collaborate with staff, teams and juniors, to share ideas and delegate tasks.

This flattened hierarchy was epitomised by the ‘4pm clinical forum’—a daily, open-invite meeting attended by staff from all directorates. This provided a shared learning environment, and a unique opportunity for collaboration and improvement at a scale, speed and transparency rarely seen in existing hospitals. To accelerate change and save time, meetings were structured around ‘safety-critical’ issues of potential patient harm. They also discussed the question ‘what do we know today that we didn’t know yesterday?’ Agreed changes were immediately delegated in person, and *in situ*, to the most appropriate stakeholder(s), and re-audited over subsequent daily meetings. As an example, during the 4pm clinical forum, an issue with the nasogastric feeding protocol was highlighted, delegated and modified by the dietetic team within 24 hours. Again, a process that would normally require weeks, if not months, in existing NHS hospitals.

## Learning hospital

To meet the demands of its rapidly changing mandate and the evolving pandemic, the NHL required an inherent capacity for improvement, agility and change. Becoming a ‘learning hospital’, where management decisions rapidly impact patient care, and *vice versa*, was therefore another early priority.^19^ To support this, a new clinical role—the ‘Bedside Learning Coordinator’—was designed and implemented.^20^ This bespoke ward-based role, created at the NHL, was also driven by the unfamiliar working environment and the need to prioritise personal protective equipment (PPE) for patient-facing staff, which at that time limited ward visits by clinical managers.^20^ The roaming BLC was responsible for *in situ* auditing of care, incident reporting and education of staff about protocol updates in real time.^20^ In practical terms, without interrupting care, BLCs would approach available ward staff, respond to queries, help those in need, complete incident forms and consider ways that patient care and staff welfare could improve. Liaising directly with clinical managers after their shifts, BLCs were crucial in shortening the quality improvement feedback loop and enabling real-time auditing, a necessity in providing true organisational agility. As an example, the BLC identified an issue with evening handover that was reported back to governance teams, delegated and amended before the next evening.

## Discussion

The NHL was a unique ‘natural experiment’ for the NHS, created as a large ICU facility in an events centre in response to the novel, contagious and pathogenic *SARS-CoV-2* coronavirus. The NHL was ready to accept patients within weeks of conception and days of construction. Although many fewer patients were admitted than originally predicted, those who were accepted experienced trajectories and outcomes equivalent to existing hospitals, a noteworthy statistic given the highly unusual circumstances of care.^6^ Beyond this, born out of urgency, necessity, workforce limitation and its unique infrastructure, the NHL provided examples of healthcare innovation that are interesting, useful and important to share.

It is necessary to emphasise that major service innovations occurred in existing hospitals, and that translating the lessons from the NHL has limitations.^21^ For example, the NHL was less vulnerable to many of the usually cited constraints to innovation—established bureaucracies, competing stakeholders and space limitations, for example.^22^ While this reduces the relevance of the NHL experience to non-pandemic times to an extent, the authors maintain that—due to the unusual circumstances and additional exceptionality—the NHL provides an important learning resource. Indeed, almost all of the above examples are not yet shared in the academic literature. With that in mind, although wide ranging and therefore difficult to contextualise fully within the literature, these lessons are important to share for their own sake—as the ‘results’ of this ‘natural experiment’—and to support healthcare academics and changemakers investigating and implementing similar changes of their own.

Interpretations and applications of the NHL experience will vary between individuals and departments. A detailed discussion of all iterations would therefore be lengthy and is beyond the scope of this article. However, to contextualise the innovations, a brief discussion of how the themes highlighted could apply to existing hospitals during non-pandemic times is beneficial. These focus on cognitive unloading, reorganised hierarchies, organisational agility, staff training and welfare.

Firstly, cognitive unloading at the NHL was a necessity during a time of acute individual and collective stress. Similar strategies could be repurposed to reduce burnout among healthcare staff, for example.^9^ At the NHL, cognitive unloading was supported by identifying staff with supply-demand mismatches, deconstructing their responsibilities and reallocating transferable duties to equally appropriate but more available staff. If required, staff duties were modified, or entirely new roles created. This allowed those at risk of cognitive overload, such as ICU nurses and consultants, to focus on their specialist non-transferable skills, as well as the piloting of innovative healthcare roles, with a new range of duties that were sometimes performed by non-clinical staff.^19 20^ Translating this fluid approach to staffing and duty allocation into existing hospitals could reduce staff burnout by supporting those in either high-demand or hard-to-fill roles.^23 24^

Secondly, at the NHL, staff education and well-being were organisational priorities second only to patient safety. This ‘double bottom line’ approach focused institutional decision-making on both patient and staff welfare. A similarly explicit realignment of organisational priorities could reduce current challenges in NHS staff recruitment, retention and well-being, for example.^25^ Indeed, improved staff welfare is known to translate into patient outcomes, institutional performance and financial efficiencies.^26^ The physical and emotional burden of having provided pandemic care is likely to exacerbate these challenges. The NHL model offers one example of how to improve staff-facing well-being support services to help benefit patients and providers.

Finally, organisational agility, as illustrated by the ‘learning hospital’, 4pm clinical forum and flattened hierarchy, had benefits and applications beyond those originally envisaged. Bureaucracy and delays in service improvement can overwhelm individual enthusiasm, and stifle innovation amongst healthcare staff.^27^ However, at the NHL, transparency in decision-making combined with a system of rapid delegated authority to the most invested stakeholder, and systems of rapid *in situ* audit, improvement and re-audit, helped encourage staff autonomy and continuous improvement. Similar measures in existing hospitals could improve staff engagement and reignite their in-built motivation for change.

## Conclusion

During the first wave of the *SARS-CoV-2* pandemic, and under close public and political scrutiny, the NHL achieved its objective of providing a safe and expandable ICU for critically ill patients with COVID-19, as an insurance policy to help mitigate the predicted capacity issues in existing NHS hospitals. 54 patients were admitted in total. With the benefit of hindsight, an ICU facility with such large potential capacity was not required. However, the NHL was a rare ‘natural experiment’ for the NHS, from which important lessons can still be learnt. In response to rapidly changing circumstances, the NHL mandate required hospital leaders, redeployed staff and non-clinical volunteers to rethink inherited wisdom—to harness their capacity for agility, creativity and change. Driven by necessity and adversity, the NHL was the setting of important, unforeseen and often emergent innovation. Although not all are directly relevant to established hospitals or departments during non-pandemic times, the practical examples, general themes and potential applications highlighted above are academically and clinically important. They could also provide the real-world examples needed to support healthcare academics and changemakers looking to investigate or implement similar changes of their own.
